# Perceived reciprocal value of health professionals’ participation in global child health-related work

**DOI:** 10.1186/s12992-017-0250-8

**Published:** 2017-05-22

**Authors:** Sarah Carbone, Jannah Wigle, Nadia Akseer, Raluca Barac, Melanie Barwick, Stanley Zlotkin

**Affiliations:** Centre for Global Child Health, The Hospital for Sick Children, 686 Bay St, Toronto, ON M5G A4 Canada

**Keywords:** Reciprocal value, Global health, Competency framework, Frugal innovation, Global partnership, Personal development, Professional development, Mutual learning

## Abstract

**Background:**

Leading children’s hospitals in high-income settings have become heavily engaged in international child health research and educational activities. These programs aim to provide benefit to the institutions, children and families in the overseas locations where they are implemented. Few studies have measured the actual reciprocal value of this work for the home institutions and for individual staff who participate in these overseas activities. Our objective was to estimate the perceived reciprocal value of health professionals’ participation in global child health-related work. Benefits were measured in the form of skills, knowledge and attitude strengthening as estimated by an adapted Global Health Competency Model.

**Methods:**

A survey questionnaire was developed following a comprehensive review of literature and key competency models. It was distributed to all health professionals at the Hospital for Sick Children with prior international work experience (*n* = 478).

**Results:**

One hundred fifty six health professionals completed the survey (34%). A score of 0 represented negligible value gained and a score of 100 indicated significant capacity improvement. The mean respondent improvement score was 57 (95% CI 53–62) suggesting improved overall competency resulting from their international experiences. Mean scores were >50% in 8 of 10 domains. Overall scores suggest that international work brought value to the hospital and over half responded that their international experience would influence their decision to stay on at the hospital.

**Conclusions:**

The findings offer tangible examples of how global child health work conducted outside of one’s home institution impacts staff and health systems locally.

**Electronic supplementary material:**

The online version of this article (doi:10.1186/s12992-017-0250-8) contains supplementary material, which is available to authorized users.

## Background

Over the past 25 years there has been heightened interest in activities and programs to improve health and health systems in low and middle-income countries [1]. In the early 1990s, the Global Health Education Consortium (GHEC), made up of a group of universities and institutions, put forward a goal to improve the health and human rights of underserved populations worldwide through improved education and training of the global health workforce [2]. Today, there are a growing number of academic health care programs (universities and academic-hospitals) that are supporting global health initiatives in education, research, and human resource capacity building. By example, the Consortium of Universities for Global Health includes 146 academic institutions and the new Consortium for Centres in Global Child Health is growing on all continents [3].

Alongside university programs targeting global health, health care institutions have also invested in a combination of humanitarian and commercial programs. Commercial programs provide treatment for visiting foreign patients and consultative services for specific sub-specialties or for hospital commissioning and administration. Humanitarian programs focus on research, education, advocacy, and capacity building. In virtually all cases, the justification underlying the global health activities is that they are consistent with the mission of the health care institutions. For example, the Boston Children’s Hospital started its Global Health program in 2013 to “create and implement innovative healthcare services that target major threats to child survival and well-being” [4]. At the Children’s Hospital of Philadelphia (CHOP) the four programs in the Department of International Medicine (International Patient Services, the Global Health Center, International Medical Education, and International Collaborations) are in line with CHOP’s mission to advance excellence in pediatric medical education, clinical service delivery and research [5]. The international program at the Hospital for Sick Children (SickKids) in Toronto, like Boston’s Children’s Hospital and CHOP, has a commercial arm for global consulting and a non-commercial arm for research, sustainable capacity building, program evaluation and advocacy. This program is consistent with the SickKids vision of, ‘healthier children, a better world’ [6]. The global health consulting components at each of these three hospitals aim to enhance local training, technology and treatment to provide world-class patient care locally, while the non-consulting components are focused on partnership development, research, education and health systems strengthening.

**Fig. 1:**
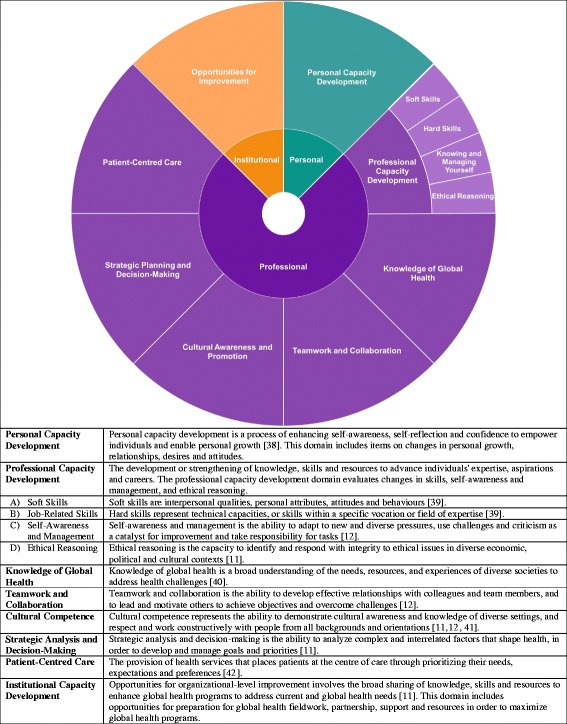
Adapted global health competency model [38–42]

The last of the Sustainable Development Goals (SDGs), Goal 17, aims to “strengthen the means of implementation and revitalize the global partnership for sustainable development” [7]. Research to date on global health collaborations and partnerships has typically focused on documenting improvements in the health outcomes of the developing countries. More recent discourse has characterized these collaborations with the notion of “reciprocal value”, namely, that the benefits go beyond strengthening the local health systems and providing world-class patient care to the children and families where the programs are implemented, and, instead, that both partners have something to learn and gain from the relationship [8]. This concept of reciprocal value highlights a greater appreciation for developing countries as partners, rather than disadvantaged consumers [9]. In addition, identifying the significance and impact of these partnerships in developed countries presents an opportunity to strengthen the partnerships and maximize the benefits for all parties involved [10]. However, to date, few studies have measured the actual reciprocal value to home institutions and to the health professionals who participate in these international activities. To explore this more closely, our study sought to estimate the perceived reciprocal value of health professionals’ participation in global child health-related work organized through SickKids. Benefits were measured in the form of skill, knowledge, and attitude strengthening as estimated by an adapted Global Health Competency Model (Fig. 1).

## Methods

This study used a survey questionnaire to evaluate the perceived value of health professionals’ participation in global health-related work. An online questionnaire was chosen as a practical method to reach all those eligible for the study. The survey questionnaire was developed following a comprehensive literature review, factor analysis and evaluation of internal consistency of domains.

The comprehensive literature search was completed prior to developing the survey, using the Queen’s University Library Catalogue (QCAT) and online search engines. We searched using the following terms: “competency development”, “reciprocal value”, “global health”, “reverse innovation”, “frugal innovation”, “global partnerships”, “mutual learning”, “personal development”, “professional development”, “international program”, and “competency framework”. Published, peer-reviewed papers written in English between 1990 and 2015, originating from any country, were searched and reviewed. The research team developed and revised the survey questions to ensure their alignment with key competency models, including the Global Health Competency Model and the WHO Global Competency Model [11, 12]. The survey initially comprised 22 questions, some with multiple sub-questions, for a total 106-items [see Additional file 1]. Questions had one of three response options: yes/no, five-point Likert scale, and open-ended. Five additional questions queried the value attributed to global health work by health professionals and senior administrators and explored the institutional support of global health work at SickKids. Informal interviews were conducted with a variety of SickKids’ health professionals having international work experience to gain further insight regarding question relevance and clarity.

Pilot testing of the survey was conducted prior to administration. Five individuals with wide experience working in developing countries were purposively selected to provide feedback on question clarity and survey length. These responses were excluded from the survey analysis. In July 2015, the web-based survey, administered through SurveyMonkey™, was emailed to 478 health professionals with a cover letter outlining the study purpose and seeking consent for participation. Respondents were given three weeks to complete the survey with one reminder. Eligibility criteria included physicians, nursing professionals (clinical, educator, nurse practitioner, etc.), research staff or allied health professionals who were currently employed at SickKids. Participants were required to have participated in professional activities, while employed at SickKids, in a country defined by the World Bank as a developing economy. Ethical approval was obtained from the Hospital for Sick Children Research Ethics Board.

The final survey tool and the proposed evidence-based framework were adapted from the aforementioned competency models and reduced using factor analysis to ensure model parsimony (see Fig. 1). The full 106-item questionnaire underwent variable reduction in three phases via factor analysis using the maximum likelihood method and a varimax rotation [13, 14]. Questions with small sample sizes (*n* < 40), those that represented overlapping concepts based on high correlation using the correlation matrix (>0.8), and questions that did not load on to a factor led to the removal of 17 items. Floor and ceiling effects, which occur when greater than 30% of responses are in the most extreme categories, were used to identify questions with potentially skewed responses [15]. This process led to the exclusion of one question, as over 70% of responses were in the ‘strongly agree’ category. Finally, eight questions with only ‘yes’ or ‘no’ responses were removed to reduce survey length and ease of completion because they acted as filters preventing some participants from completing all questions. The final reciprocal value survey was comprised of 80-items, including demographic information (7 items), institutional value (5 items), and 68-items distributed across ten domains capturing personal, professional and institutional capacity development. Results from the institutional development domain were not included in this report since it was not directly related to reciprocal value of global child health work for individuals.

Descriptive statistics outlined key characteristics of the survey sample by age, sex, type and length of employment, and number and length of global health work-trips (Table 1). Potential sampling bias was assessed by comparing demographic results of the survey sample against the target population of individuals who received the survey but did not respond (Table 2). Domain composite scores were calculated based on the mean of responses by item (coded as strongly disagree = 0; disagree = 1; neutral = 2; agree = 3; strongly agree = 4), and displayed as a proportion out of the maximum possible domain score. A score of 0 represented negligible or limited value gained as a result of global health efforts, and a score of 100 indicated that individuals felt strongly that their capacity in a particular domain was improved as a result of their international experiences. Analysis of the mean proportion, standard deviation and 95% confidence intervals were calculated for each domain composite score (Table 3). The distribution of responses was analyzed by tertile to determine the proportion of responses that responded in the low (<33.3), mid (33.3–66.6) or high (>66.6) range.

**Table 1:** Demographic characteristics of participants (*N* = 156)

Variables		*N*	Percentage (%)
Total	Number of surveys sent	478	100
	Responses	161	34
	Sample for analysis	156	33
Sex	Male	25	16
	Female	131	84
Age	25 - < 35 years	25	16
	35–50 years	72	46
	>50 years	59	38
Occupation	Physician	27	17
	Nurse	65	42
	Allied Health Professional	37	24
	Researcher	11	7
	Other	16	10
Length of Employment	<5 years	20	13
	5–10 years	32	21
	10–20 years	59	38
	>20 years	45	29
Number of times participated in international work through SickKids	1 time	53	34
	2–3 times	50	32
	4–5 times	27	17
	6 or more	25	16
Longest amount of time spent on a single visit to a developing country as a SickKids employee	<1 week	20	13
	1- < 4 weeks	84	54
	1–3 months	31	27
	>3 months	10	7

**Table 2:** Assessment of sampling bias

Variable		Participant breakdown *N*, (%)	Overall survey breakdown, *N* (%)
Sex	Male	25 (16%)	114 (24%)
	Female	131 (84%	364 (76%)
Occupation	Physician	27 (17%)	77 (16%)
	Nurse	65 (42%)	208 (44%)
	Allied Health Professional	37 (24%)	73 (15%)
	Researcher	11 (7%)	33 (7%)
	Other	16 (13%)	87 (18%)

**Table 3:** Overall domain scores

Domain	Measure of improvement		Responses (by Tertile)			
	Mean Score (S.D)	95% Confidence Interval	Lowest *N* (%)	Med *N* (%)	Highest, *N* (%)	Goodness-of-Fit (*p*-value)
Knowledge of global health	79 (21)	75–82	7 (5%)	21 (14%)	128 (82%)	<0.001
Cultural awareness & promotion	63 (33.0)	57–68	30 (22%)	17 (12%)	92 (66%)	<0.00
Teamwork & Collaboration	51 (29)	46–56	44 (30%)	47 (32%)	56 (38%)	0.45
Personal Capacity Development	76 (15)	73–78	1 (1%)	40 (26%)	115 (73%)	<0.000
Soft Skills	53 (30)	49–58	46 (30%)	39 (25%)	71 (46%)	0.004
Job-related skills	44 (28)	39–48	49 (31%)	77 (49%)	30 (19%)	<0.000
Self-management & awareness	56 (29)	52–61	39 (25%)	44 (28%)	73 (47%)	0.002
Ethical reasoning	47 (28)	42–51	44 (35%)	50 (40%)	32 (25%)	0.14
Patient-centred care	52 (34)	46–57	53 (34%)	33 (21%)	70 (45%)	0.001
Strategic analysis & decision-making	54 (31)	49–59	43 (28%)	46 (30%)	67 (43%)	0.037

Data was analyzed to assess competencies and benefits experienced through global health work by staff occupation, length of employment and the number of global health placements. Further investigation of these characteristics was informed by similar groupings reported in the literature. Analysis of results by occupation focused on physicians, nurses, and allied health professionals. Results for ‘researchers’ and ‘others’ were not analyzed, as researchers represented a small sample size (*n* = 11), and occupations included in the ‘other’ category ranged significantly from senior management and administration, to computer and technical support staff. One-way ANOVA was used to assess the variance in mean scores for domains by type of occupation, length of employment, and number of global health trips. Multiple comparisons were conducted using post-hoc Tukey’s test to analyze which sub-groups were statistically significant in their responses. The distribution in responses by sub-groups was assessed using tertile analysis by occupation, length of employment and number of times travelled for global health work. Chi-square tests were used to test proportions across the subgroups (goodness of fit) except when assumptions of chi-square test were violated in which case Fishers’ Exact Test was used (Table 3). The type 1 error rate was controlled at 0.05 and all statistical analyses were conducted using SPSS 23.0. Answers to the open-ended questions were analysed by two of the authors (SC, RB) using qualitative content analysis [16, 17].

## Results

The survey was sent to 478 participants and achieved a moderate response rate of 34%, which is within the normal range for web surveys [18]. Among the 161 completed surveys, 5 responses were excluded from the final analysis for failing to meet the original eligibility criteria and 26 questions were removed for reasons outlined in the methods section. Participant demographic characteristics are shown in Table 1. The majority of respondents were nurses, while 17% were physicians. Most respondents spent between 1 and 4 weeks on their international assignments and were long-term employees of the hospital. Additional file 2 provides a complete list of the countries where participants had worked. Respondents were similar in gender and occupation to non-respondents (Table 2).

**Fig. 2:**
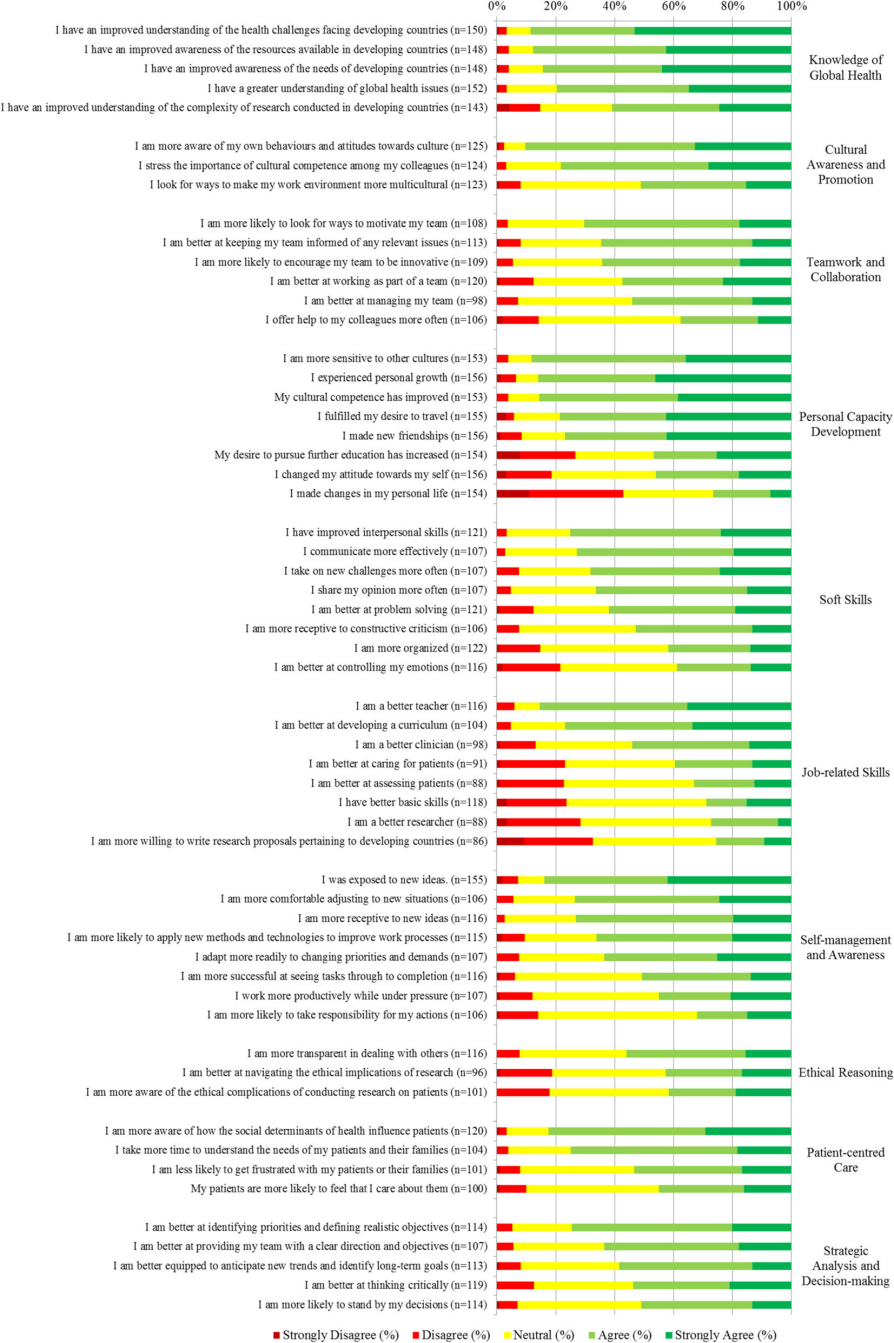
Participant responses to likert-scale questions organized by domain

Table 3 shows overall mean scores by domain and the distribution of responses according to low, middle and high tertiles. Mean scores were >50% in 8 of the 10 domains. Jobs-related skills and ethical reasoning were the only domains where the mean scores were <50%. The domains with the highest mean proportions were knowledge of global health, personal capacity development and cultural awareness and promotion. There were statistically significant goodness-of-fit differences (*p* < 0.05) in all domains, with the exception of teamwork and collaboration, and ethical reasoning. Domains were further broken down and analyzed according to individual questions (Fig. 2). The spectrum of dark green, to dark red, illustrated in Fig. 2 shows participant responses ranging from ‘strongly agree’ to ‘strongly disagree’. Overall, these findings demonstrate that for most domains, participants reported substantial competency development resulting from their global work.

Analysis of overall domain scores was stratified into three subgroups including: occupation, length of employment and number of global health experiences (Tables 4, 5 and 6). The first analysis compared physicians, nurses and allied health professionals (Table 4). In all domains physicians had lower mean scores than both nurses and allied health professionals. Differences were statistically significant (*p* < 0.05) for five of the ten domains. In particular, there were significant differences in mean scores for personal capacity development, soft skills, job-related skills, self-management and awareness, and strategic analysis and decision-making.

**Table 4:** Subgroup analysis of overall mean scores according to occupation

Domain	Physician *n = 27*	Nurse *n = 65*	Allied Health Professionals *n = 37*	One-way ANOVA (*p*-value)
Knowledge of global health	70 (24)*	81 (19)	77 (21)	0.072
Cultural awareness & promotion	55 (36)	66 (31)	62 (32)	0.37
Teamwork & Collaboration	41 (31)	56 (28)	47 (27)	0.065
Personal Capacity Development	68 (15)^a^	77 (15)^b^	78 (14)^b^	0.011
Soft Skills	35 (30)^a^	57 (29)^b^	59 (29)^b^	0.002
Job-related skills	35 (32)^a^	51 (26)^b^	50 (24)^b^	0.028
Self-management & awareness	43 (29)^a^	59 (28)^b^	59 (30)^b^	0.043
Ethical reasoning	40 (26)	51 (27)	45 (27)	0.20
Patient-centred care	52 (33)	61 (34)	58 (31)	0.49
Strategic analysis & decision-making	41 (32)^a^	59 (29)^b^	54 (32)^b^	0.045

*Mean (SD). Professions with non-matching superscripts are significantly different (Tukey HSD)

**Table 5:** Subgroup analysis of overall mean scores according to length of employment

Domain	Length of Employment				One-Way ANOVA (*p*-value)
	<5 years *n = 20*	5–10 years *n = 32*	10–20 years *n = 59*	>20 years *n = 45*	
Knowledge of global health	83 (15)*	78 (21)	78 (22)	78 (22)	0.83
Cultural awareness & promotion	55 (37)	65 (31)	62 (36)	66 (29)	0.67
Teamwork & Collaboration	48 (33)	56 (27)	52 (31)	48 (28)	0.62
Personal Capacity Development	73 (14)	81 (12)	75 (15)	73 (16)	0.12
Soft Skills	51 (34)	65 (25)	52 (34)	48 (26)	0.09
Job-related skills	32 (28)	46 (25)	46 (29)	44 (26)	0.21
Self-management & awareness	51 (32)	65 (23)	56 (32)	52 (28)	0.18
Ethical reasoning	39 (33)	52 (25)	45 (30)	48 (25)	0.45
Patient-centred care	30 (35)^a^	52 (32)^b^	55 (36)^b^	56 (30)^b^	0.025**
Strategic analysis & decision-making	49 (34)	61 (28)	55 (33)	49 (29)	0.29

*Mean (SD). Lengths of employment with non-matching superscripts are significantly different (Tukey HSD)

**P = 0.014 for the linear-by-linear association test

**Table 6:** Subgroup analysis of overall mean scores according to number of international work experiences

Domain	Number of international work experiences			One-Way ANOVA (*p*-value)
	1 time *n = 53*	2–3 *n = 50*	4+ *n = 52*	
Knowledge of global health	79 (17)	77 (21)	80 (24)	0.73
Cultural awareness & promotion	60 (34)	63 (33)	64 (33)	0.79
Teamwork & Collaboration	50 (29)	47 (30)	57 (28)	0.21
Personal Capacity Development	76 (13)	74 (16)	76 (16)	0.77
Soft Skills	55 (31)	49 (32)	56 (29)	0.44
Job-related skills	44 (27)	41 (27)	45 (29)	0.79
Self-management & awareness	57 (29)	52 (30)	60 (28)	0.36
Ethical reasoning	46 (28)	43 (28)	52 (28)	0.26
Patient-centred care	53 (36)	50 (33)	52 (34)	0.92
Strategic analysis & decision-making	53 (31)	51 (32)	57 (30)	0.62

Table 5 shows participant responses according to length of employment at SickKids. Only patient-centred care was significantly different among participants with <5 years versus more experience. The final subgroup analysis examined whether the frequency of individual international work visits influenced self-perceived competency development (Table 6). Participants who had travelled on a single occasion were compared with those who had travelled a moderate amount (2–3 occasions), and those who had travelled frequently (>4 occasions). There were no differences in mean scores among groups, suggesting that participants reported the same competency development regardless of the number of occasions they had worked internationally.

Table 7 highlights respondent perceptions of whether their global work was valued within the hospital and brought value to the hospital. Overall scores suggest that participants believed their work was valued by others within the hospital, particularly senior leaders (61%) and was of value to the hospital. A high proportion of participants also felt that their global work had positively impacted the SickKids brand and over half responded that their international experience would influence their continuing to work at SickKids.

**Table 7:** Perceptions of value to the hospital. These questions were not included in the domains

Questions	Not at all (%)	Somewhat (%)	Neutral (%)	Mostly (%)	Absolutely (%)
Do you think that your work overseas is valued by your colleagues? (*n* = 135)	10	21	14	38	17
Do you think that your work overseas is valued by senior leaders of the hospital? (*n* = 135)	5	18	16	41	20
Do you think that your work overseas is valued by the hospital foundation? (*n* = 135)	10	15	30	29	16
Are you more likely to continue working at SickKids due to the availability of international experiences? (*n* = 135)	15	8	25	21	31
Do you think that your international experience has had a positive impact on the SickKids brand in Canada? (*n* = 135)	5	4	19	22	50

Responses to the open-ended questions were in line with the quantitative data and revealed that participants greatly valued their international work experiences, desired to participate again and had a variety of suggestions to improve the experience for future participants [see Additional file 3]. Many participants reflected positively on their global work experiences, suggesting that they contributed to strengthening their professional skills (“I think it definitely strengthened my skills and boosted my professional confidence”), offered an opportunity for professional collaboration or networking: (“It gave me the opportunity to meet and work with SickKids staff who I would otherwise have never met”), and enhanced their motivation to pursue new learning opportunities and their sense of fulfillment in their current roles (“I decided to go back to school – Masters in Health Management – due to my experience..)”. Several participants also identified innovative ideas or technologies to which they had been exposed during their global work (eg. tool for computing nutrient intake; ensuring that the curriculum included a cultural competency component) and talked about changes they implemented in their local workplace that were inspired or informed by these global work experiences. These innovations were frequently in the areas of care provision, teaching, research and communication. For example, one participant noted that they had been introduced to a newborn passport in Tanzania that “ensure[d] that all receive access to essential care”.

## Discussion

Our results demonstrate that health professionals’ participation in global child health-related work in developing countries is associated with positive competency development as measured by improvements in perceived knowledge of global health, personal capacity building, cultural awareness and promotion, skills, knowledge and attitude strengthening. Additionally, more than half of the participants responded that their international work had a positive impact on the SickKids brand locally, and importantly, that they are more likely to continue working at SickKids due to the continuing availability of international experiences. The findings offer tangible examples of how global health work outside of one’s home institution impacts local health systems on the individual and institutional levels and expands our understanding of the value of global health partnerships. Additionally, this kind of research is consistent with SDG Goal 17 that focuses on strengthening global partnerships for sustainable development [7].

Positive competency development among SickKids health professionals is consistent with findings from similar studies involving volunteers [19]. Participants reported the most value in the areas of knowledge of global health, cultural awareness and promotion, and personal capacity development. The perceived improvements in these areas may be in part due to the profound effects of cultural immersion, as all participants worked and lived abroad [20–23].

There is ongoing debate over the value gained by health professionals who partake in short (1–4 weeks) versus long-term (>4 weeks) global work or placements [24]. Short-term visits may impose a burden on the host country and some researchers argue that long-term experiences are required to maximize impact and sustain learning over time [25–29]. In contrast, others have found that short-term visits to developing countries are also of benefit to the health professional (from a high income country) [30, 31]. Our findings suggest that both short- and long-term visits have a substantial positive impact on health professionals. Furthermore, respondents who participated in a single, short-term visit reported comparable competency development with those who travelled for longer periods or on multiple separate occasions. This perceived impact did not appear to deteriorate over time.

Our findings are consistent with claims that ‘north-south’ projects support reverse or ‘frugal’ innovations [32]. More than 80% of respondents identified that they had been exposed to new ideas and 65% said that they were more likely to apply these new methods and technologies to improve work processes at SickKids. Novel communication technologies, previously noted in other studies, were among the most frequently reported innovations [33]. There was a strong sense that work abroad improved personal capacity development (76% agree or strongly agree), especially in cultural competence and sensitivity. More than 50% of participants indicated that they would stress the importance of cultural competence among their colleagues in their local workplace and look for ways to make their work environment more multicultural. Some participants noted that their improved awareness and acceptance of cultural diversity was shared within their hospital units through the development of improved multicultural training resources. This development is especially important in Toronto, now considered one of the most culturally diverse large cities in the world [34]. These results were similar independent of years of service at SickKids and number of visits abroad.

In addition to the acquisition of new knowledge and innovations, global work also encouraged personal development among participants and in many cases created greater job-satisfaction and loyalty to SickKids. Participants reported becoming champions of global health within their home institution, encouraging greater involvement in global work among their colleagues and improving resources for patients. Even among those who did not implement any changes at SickKids, it was clear that the vast majority of participants enjoyed their experience (92%), and were more likely to continue working at SickKids due to the availability of global work (60%). These findings suggest that global work may help health care institutions retain experienced health professionals and improve morale. Furthermore, these findings fit with the key role played by practitioners’ knowledge and beliefs and, more generally, practitioners’ characteristics - which is one of the five domains in the Consolidated Framework for Implementation Research - and has been shown to be related to implementation success in health and global health [35–37].

Despite efforts to include a diverse sample of occupations, experiences and partnerships within the data set, the study had several limitations. Inclusion criteria were restricted to staff at SickKids who had worked internationally through SickKids supported programs, thus excluding those who volunteered on their own time or had previous work experience in global health. The resulting sample size was relatively small and the majority of respondents were female and nurses. Though respondents were representative of the overall surveyed population, findings may not be generalizable beyond SickKids. Previous experience in and exposure to global work outside of SickKids may have inadvertently influenced individuals’ responses. The administration of a common survey among such a diverse population presented a unique challenge during analysis due to the variation in sample size for each question, especially those questions related to job-related skills. For example, participants occupying a clinical role may have reported greater improvements in their patient-care skills than participants who had limited contact with patients.

## Conclusion

The findings of this study are of value to academic health care institutions with an interest or stake in global collaborations. Our findings demonstrated the substantial reciprocal competency development that occurs among health professionals working globally, as well as the extent to which this learning may impact home institutions. We believe that involvement in global health collaborations presents a wide range of potential benefits to developing countries, but is also of value to ‘home institutions’ and their staff participating in these global child health opportunities.

## Additional files

Additional file 1: Survey - Determining the reciprocal value of your global health experience. This file contains the finalized survey tool used to collect data in this study. (DOCX 137 kb)

Additional file 2: List of countries visited. This file contains a complete list of countries where respondents had previously worked, as well as the number of respondents who worked at each location. (DOCX 72 kb)

Additional file 3: Summary of participant responses to open-ended survey questions. This file contains a summary of all participant responses to open-ended survey questions. (DOCX 128 kb)

## Abbreviation

SickKids: The hospital for sick children

## References

[1] Easterbrook P (2011). Institutional partnerships in global health. Clin Med.

[2] World Health Organization: Global Health Education Consortium. 2016. http://www.who.int/workforcealliance/members_partners/member_list/ghec/en/. Accessed 27 July 2016.

[3] Consortium of Universities for Global health: Member Institutions. 2016. http://www.cugh.org/members. Accessed 27 July 2016.

[4] Boston Children’s Hospital: For Health Professionals, International Health Services. http://www.childrenshospital.org/international/for-health-professionals. Accessed 27 July 2016.

[5] The Children’s Hospital of Philadelphia: Our Mission.CHOP. 2016. http://www.chop.edu/about-us/our-mission#.V63xa2XXBEc. Accessed 27 July 2016.

[6] The Hospital for Sick Children: Vision, mission and values. Sickkids. 1999. http://www.sickkids.ca/AboutSickKids/who-we-are/Vision-Mission-Values/index.html. Accessed 27 July 2016.

[7] United Nations: Sustainable Development Goals. 2015. https://sustainabledevelopment.un.org/?menu=1300#. Accessed 17 Apr 2017.

[8] Busse H, Azazh A, Tupesis JP, et al. Creating change through collaboration: a twinning partnership to strengthen emergency medicine at addis ababa University/Tikur anbessa specialized hospital—a model for international medical education partnerships. Acad Emerg Med. 2013; doi:10.1111/acem.12265. 10.1111/acem.12265 24341587

[9] Crisp N (2010). Turning the world upside down: the search for global health in the twenty-first century.

[10] Botenbal M. Differences in learning practices and values in north-south city partnerships: towards a broader understanding of mutuality. Public Admin Develop. 2013; doi:10.1002/pad.1622.

[11] Ablah E, Biberman D, Weist E, Buekens P, Bentley M, Burke D (2014). Improving global health education: development of a global health competency model. AmJTrop Med Hyg.

[12] World Health organization: WHO Global Competency model. 2010. http://www.who.int/employment/competencies/WHO_competencies_EN.pdf. Accessed 3 June 2015.

[13] Yong AG, Pearce S (2013). A beginner’s guide to factor analysis: focusing on exploratory factor analysis. Tutor Quant Methods Psychol.

[14] Fabrigar LR, Wegener DT, MacCallum RC, Strahan EJ. Evaluating the use of exploratory factor analysis in psychological research. Psychol Methods. 1999; doi:10.1037/1082-989X.4.3.272.

[15] Veras M, Pottie K, Welch V, Labonte R, Eslava-Schmalbach J, Borkhoff C (2012). Reliability and validity of a new survey to assess global health competencies of health professionals. Glob J Health Sci.

[16] Morgan DL. Qualitative content analysis: a guide to paths not taken. Qual Health Res. 1993; doi:10.1177/104973239300300107. 10.1177/104973239300300107 8457790

[17] Elo S, Kyngas H. The qualitative content analysis process. J Adv Nurs. 2008; doi:10.1111/j.1365-2648.2007.04569.x. 10.1111/j.1365-2648.2007.04569.x 18352969

[18] Shih TH, Xitao F. Comparing response rates from web and mail surveys: a meta-analysis. Field Methods. 2008; doi:10.1177/1525822X08317085.

[19] Busse H, Aboneh E, Tefera G (2014). Learning from developing countries in strengthening health systems: an evaluation of personal and professional impact among global health volunteers at Addis Ababa University’s Tikur Anbessa specialized hospital (Ethiopia). Glob Health.

[20] Kollar S, Ailinger R (2002). International clinical experiences. Nurse Educ.

[21] Larsen RL (2011). Effectiveness of cultural immersion and culture classes for enhancing nursing students’ transcultural self-efficacy. J Nurs Educ.

[22] Mixer S. Use of the culture care theory and ethnonursing method to discover how nursing faculty teach culture care. Contemp Nurse. 2008; doi:10.5172/conu.673.28.1-2.23. 10.5172/conu.673.28.1-2.23 18844555

[23] Halter M, Grund F, Fridline M, See S, Young L, Reece C. Transcultural self-efficacy perceptions of baccalaureate nursing students. J Transcult Nurs. 2014; doi:10.1177/1043659614526253. 10.1177/1043659614526253 24841469

[24] Button L, Green B, Tengnah C, Johansson I, Baker C. The impact of international placements on nurses' personal and professional lives: literature review. J Adv Nurs. 2005; doi:10.1111/j.1365-2648.2005.03395.x. 10.1111/j.1365-2648.2005.03395.x 15811111

[25] Cameron D. Working internationally. Phys Occup Ther Pediatr. 2008; doi:10.1080/01942630802031792. 10.1080/01942630802031792 18846891

[26] Frenk J, Chen L, Bhutta Z, Cohen J, Crisp N, Evans T, et al. Health professionals for a new century: transforming education to strengthen health systems in an interdependent world. Lancet. 2010; doi:10.1016/S0140-6736(10)61854-5. 10.1016/S0140-6736(10)61854-5 21112623

[27] Rhee D, Heckman J, Chae S, Loh L (2014). Comparative analysis: potential barriers to career participation by north american physicians in global health. Int J Family Med.

[28] McElmurry B, Misner S, Buseh A. Minority international research training program: global collaboration in nursing research. J Prof Nurs. 2003; doi:10.1053/jpnu.2003.10. 10.1053/jpnu.2003.10 12649816

[29] Zorn C. The long-term impact on nursing students of participating in international education. J Prof Nurs. 1996; doi:10.1016/S8755-7223(96)80056-1. 10.1016/s8755-7223(96)80056-1 8632096

[30] Inglis A, Rolls C, Kristy S. The impact on attitudes towards cultural difference of participation in a health focused study abroad program. Contemp Nurse. 2000; doi:10.5172/conu.2000.9.3-4.246. 10.5172/conu.2000.9.3-4.246 11855033

[31] Curtin A, Martins D, Schwartz-Barcott D, DiMaria L, Ogando B. Development and evaluation of an international service learning program for nursing students. Public Health Nurs. 2013; doi:10.1111/phn.12040. 10.1111/phn.12040 24579714

[32] Syed S, Dadwal V, Martin G. Reverse innovation in global health systems: towards global innovation flow. Glob Health. 2013; doi:10.1186/1744-8603-9-36. 10.1186/1744-8603-9-36 PMC3846449 23992598

[33] Howitt P, Darzi A, Yang G, et al. Technologies for global health. Lancet. 2012; doi:10.1016/S0140-6736(12)61127-1.

[34] Statistics Canada. 2011 National Household Survey: immigration, citizenship, place of birth, ethnicity, visible minorities, religion and aboriginal peoples. Toronto; 2011. https://www1.toronto.ca/city_of_toronto/social_development_finance__administration/files/pdf/nhs_backgrounder.pdf. Accessed 30 July 2016

[35] Damschroder L, Aron D, Keith R, Kirsh S, Alexander J, Lowery J. Fostering implementation of health services research findings into practice: a consolidated framework for advancing implementation science. Implement Sci. 2009; doi:10.1186/1748-5908-4-50. 10.1186/1748-5908-4-50 PMC2736161 19664226

[36] Connell LA, McMahon NE, Watkins CL, Eng JJ. Therapists’ use of the graded repetitive arm supplementary program (GRASP) intervention: a practice implementation survey study. Phys Ther. 2014; doi:10.2522/ptj.20130240. 10.2522/ptj.20130240 PMC4016677 24505098

[37] Barwick M, Barac R, Zlotkin S. Evaluation of effective implementation of exclusive breastfeeding in Ethiopia and Mali using the consolidated framework for implementation research. The Hospital for Sick Children, Canada. http://www.can-mnch.ca/wp-content/uploads/2015/05/EBF-Research-Report-FINAL-July-29-2015.pdf. Accessed 27 July 2016.

[38] Gadd G. Career enhancement through personal development. Vet Nurs J. 2012; doi:10.1111/j.2045-0648.2012.00183.x.

[39] Robles G. Executive perceptions of the top 10 soft skills needed in todays workplace. Bus Comm Quart. 2012; doi:10.1177/1080569912460400.

[40] Koplan JP, Bond TC, Merson MH, et al. Towards a common definition of global health. Lancet. 2009; doi:10.1016/S0140-6736(09)60332-9. 10.1016/S0140-6736(09)60332-9 PMC9905260 19493564

[41] Campinha-Bacote J. The process of cultural competence in the delivery of healthcare services: a model of care. J Transcult Nurs. 2002; doi:10.1177/10459602013003003. 10.1177/10459602013003003 12113146

[42] Dambha H, Griffin S, Kinmonth A. Patient-centred care in general practice. InnovAiT. 2014; doi:10.1177/1755738014544482.

