# Supplementary material for: Perceived reciprocal value of health professionals’ participation in global child health-related work
Source: Global Health. 2017 May 22;13:27. doi: 10.1186/s12992-017-0250-8 (PMC5441071; doi:10.1186/s12992-017-0250-8)
Supplement: Supplementary file 1 — Survey - Determining the reciprocal value of your global health experience. This file contains the finalized survey tool used to collect data in this study. (DOCX 137 kb) [file 12992_2017_250_MOESM1_ESM.docx]

**Appendix A: Survey - Determining the reciprocal value of your global health experience**

**Introduction**

This survey is designed to allow you to reflect upon your international work experience facilitated by SickKids International or the SickKids Centre for Global Child Health. Your participation will allow the Investigators to better assess the value of global health work to SickKids, and ensure that the future program participants obtain the most from their international experiences.

Please answer the following questions to the best of your ability. You may provide as little or as much detail as you like. This survey is anonymous and will not be used to identify you in any way.

**Background Information**

1. What is your gender? *(select one option)*

- Male
- Female
- Other

2. What is your age? *(select one option)*

- < 25 years
- 25 - <35 years
- 35 - 50 years
- > 50 years

3. How long have you been working at SickKids? (*select one option)*

- <5 years
- 5-10 years
- 10-20 years
- >20 years

4. What is your current role at SickKids? *(select one option)*

- Physician
- Registered Nurse
- Nurse Educator
- Researcher
- Allied Health Professional
- Other (*please* *specify*)

5. How many times have you participated in international work run through SickKids? *(select one option)*

- 1
- 2-3
- 4-5
- 6 or more
- Never

6. What is the longest amount of time you have spent on a single international visit through SickKids?? *(select one option)*

- < 1 week
- 1 - < 4 weeks
- 1 - 3 months
- > 3 months
- I have never worked internationally for SickKids.

7. Please list the countries you have visited during your international work for SickKids. (*list all that apply)*

8. Have you ever been involved in an international health-related program outside of SickKids?

- Yes
- No

9. Is international travel an expected part of your current work at SickKids?

- Yes
- Sometimes
- No

**Personal Impact**

10. To what extent did you enjoy your most recent international experience facilitated by SickKids?

- Absolutely
- Mostly
- Neutral
- Somewhat
- Not at all

11. To what extent do you feel you personally benefitted from your international experience(s)?*(Select the category that best reflects your experience)*

|  | Strongly Disagree (1) | Disagree  (2) | Neutral  (3) | Agree  (4) | Strongly Agree (5) | N/A |
| --- | --- | --- | --- | --- | --- | --- |
| I fulfilled my desire to travel. | 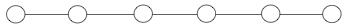 | | | | | |
| I experienced a new culture. | 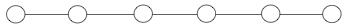 | | | | | |
| I made new friendships. | 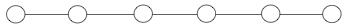 | | | | | |
| I changed my attitude towards my self. | 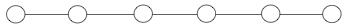 | | | | | |
| I learned or strengthened skills. | 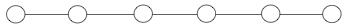 | | | | | |
| I experienced personal growth. | 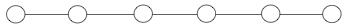 | | | | | |
| I made changes in my personal life. | 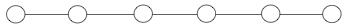 | | | | | |
| I was exposed to new ideas. | 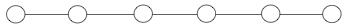 | | | | | |
| My desire to pursue further education has increased. | 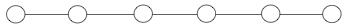 | | | | | |

**Professional Impact**

12. In what ways has your international experience increased your knowledge of global health?

*(Select the category that best reflects your experience)*

|  | Strongly Disagree (1) | Disagree  (2) | Neutral  (3) | Agree  (4) | Strongly Agree  (5) | N/A |
| --- | --- | --- | --- | --- | --- | --- |
| I am more sensitive to other cultures. | 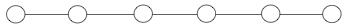 | | | | | |
| I have an improved understanding of the health-challenges facing developing countries. | 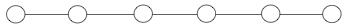 | | | | | |
| I have an improved awareness of the needs of developing countries. | 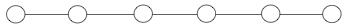 | | | | | |
| I have an improved awareness of the strengths of developing countries. | 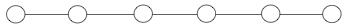 | | | | | |
| I have an improved awareness of the resources available in developing countries. | 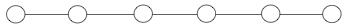 | | | | | |
| I have a greater understanding of global health issues. | 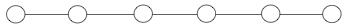 | | | | | |
| My cultural competence has improved. | 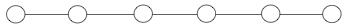 | | | | | |
| I have an improved understanding of the complexity of research conducted in developing countries. | 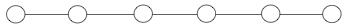 | | | | | |

13. Do you feel more confident in your work at SickKids as a result of your overall international experience?

- Yes (go to question 13.1)
- No

13.1 To what extent has your improved confidence impacted your work at SickKids?

*(Select the category that best reflects your experience)*

|  | Strongly Disagree (1) | Disagree  (2) | Neutral  (3) | Agree  (4) | Strongly Agree  (5) | N/A |
| --- | --- | --- | --- | --- | --- | --- |
| I share my opinion more often. | 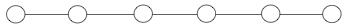 | | | | | |
| I communicate more effectively. | 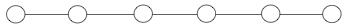 | | | | | |
| I encourage colleagues to share their views more often. | 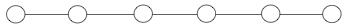 | | | | | |
| I work more productively while under pressure. | 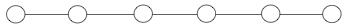 | | | | | |
| I am more receptive to constructive criticism. | 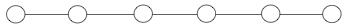 | | | | | |
| I am more successful assessing patients. | 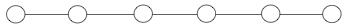 | | | | | |
| I deliver better care to patients. | 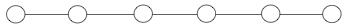 | | | | | |
| I take on new challenges more often. | 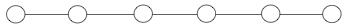 | | | | | |
| I have adopted new responsibilities. | 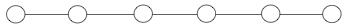 | | | | | |
| I adapt more readily to changing priorities and demands. | 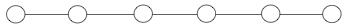 | | | | | |
| I offer help to my colleagues more often. | 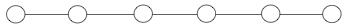 | | | | | |
| I am more likely to take responsibility for my actions. | 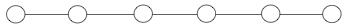 | | | | | |
| I am better at navigating the ethical implications of research. | 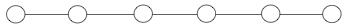 | | | | | |
| I am more willing to write research proposals pertaining to developing countries. | 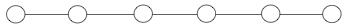 | | | | | |
| I am more comfortable adjusting to new situations. | 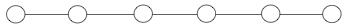 | | | | | |

14. Has your overall international experience overseas improved your leadership abilities?

- Yes (go to question 14.1)
- No

14.1 To what extent has your improved capacity for leadership impacted your work at SickKids?

*(Select the category that best reflects your experience)*

|  | Strongly Disagree (1) | Disagree  (2) | Neutral  (3) | Agree  (4) | Strongly Agree  (5) | N/A |
| --- | --- | --- | --- | --- | --- | --- |
| I am better at keeping my team informed of any relevant issues. | 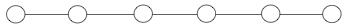 | | | | | |
| I am more successful at seeing tasks through to completion. | 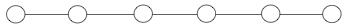 | | | | | |
| I am more receptive to new ideas. | 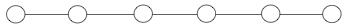 | | | | | |
| I am more likely to apply new methods and technologies to improve work processes. | 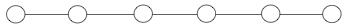 | | | | | |
| I am better at managing my team. | 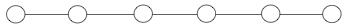 | | | | | |
| I am more transparent in dealing with others. | 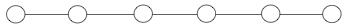 | | | | | |
| I am more likely to act consistently between expressed principles and behaviours. | 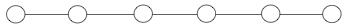 | | | | | |
| I am more likely to stand by my decisions. | 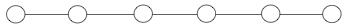 | | | | | |
| I am better at providing my team with a clear direction and objectives. | 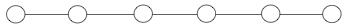 | | | | | |
| I am more likely to look for ways to motivate my team. | 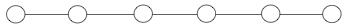 | | | | | |
| I am better at identifying priorities and defining realistic objectives. | 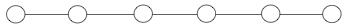 | | | | | |
| I am better equipped to anticipate new trends and identify long-term goals. | 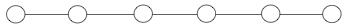 | | | | | |
| I am more likely to encourage my team to be innovative. | 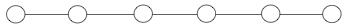 | | | | | |

15. Has your overall international experience improved your cultural competence?

- Yes (go to question 15.1)
- No

15.1 To what extent has your improved cultural competence impacted your work at SickKids?

*(Select the category that best reflects your experience)*

|  | Strongly Disagree (1) | Disagree  (2) | Neutral  (3) | Agree  (4) | Strongly Agree  (5) | N/A |
| --- | --- | --- | --- | --- | --- | --- |
| I take more time to understand the needs of my patients and their families. | 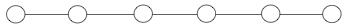 | | | | | |
| I make more of an effort to make my patients and their families feel comfortable. | 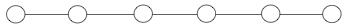 | | | | | |
| I make more of an effort to learn about my patients and their families. | 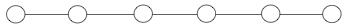 | | | | | |
| I am more likely to treat each patient as unique. | 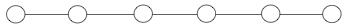 | | | | | |
| I stress the importance of cultural competence among my colleagues. | 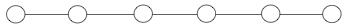 | | | | | |
| I look for ways to make my work environment more multicultural. | 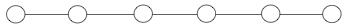 | | | | | |
| I am less likely to get frustrated with my patients or their families. | 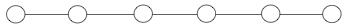 | | | | | |
| My patients are more likely to feel that I care about them. | 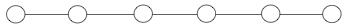 | | | | | |
| I am more sensitive to the role of patients and their families in research. | 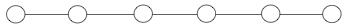 | | | | | |
| I am more aware of the ethical complications of conducting research on patients. | 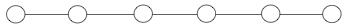 | | | | | |
| I am more aware of how the social determinants of health influence patients. | 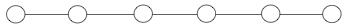 | | | | | |
| I am more aware of my own behaviours and attitudes towards culture. | 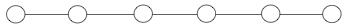 | | | | | |

16. Did you learn or improve upon any skills related to your work during your overall international experience? *(These skills may be clinical, academic, leadership, interpersonal, or in any other domains)*

- Yes (go to question 16.1, 16.2)
- No (go to question 16.2)

16.1 To what extent did you learn or strengthen the following skills during your international work experience? *(Select the category that best reflects your experience)*

|  | Strongly Disagree (1) | Disagree  (2) | Neutral  (3) | Agree  (4) | Strongly Agree  (5) | N/A |
| --- | --- | --- | --- | --- | --- | --- |
| I am a better clinician. | 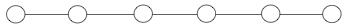 | | | | | |
| I am a better researcher. | 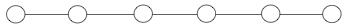 | | | | | |
| I am a better teacher. | 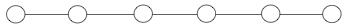 | | | | | |
| I have improved interpersonal skills. | 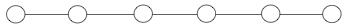 | | | | | |
| I am a better communicator. | 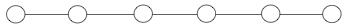 | | | | | |
| I am better at assessing patients. | 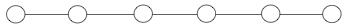 | | | | | |
| I am better at caring for patients. | 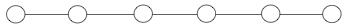 | | | | | |
| I am better at controlling my emotions. | 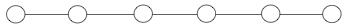 | | | | | |
| I am better at problem solving. | 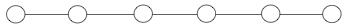 | | | | | |
| I am more organized. | 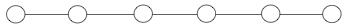 | | | | | |
| I am better at working as part of a team. | 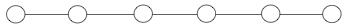 | | | | | |
| I am better at thinking critically. | 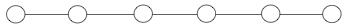 | | | | | |
| I have better basic skills (reading, writing etc). | 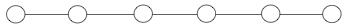 | | | | | |
| I am better at developing a curriculum. | 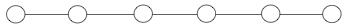 | | | | | |

16.2 How could your international experience be improved to facilitate greater skill development among SickKids employees? *(Select the category that best reflects your experience)*

|  | Strongly Disagree (1) | Disagree  (2) | Neutral  (3) | Agree  (4) | Strongly Agree  (5) | N/A |
| --- | --- | --- | --- | --- | --- | --- |
| There should be stronger support before, during or after overseas visits. | 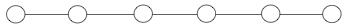 | | | | | |
| There should be more comprehensive training before travelling overseas. | 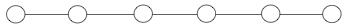 | | | | | |
| Overseas visits should be longer. | 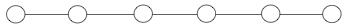 | | | | | |
| There should be more collaboration between partners. | 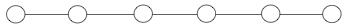 | | | | | |
| There should be more opportunities to practice skills. | 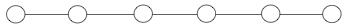 | | | | | |
| There should be more opportunities to provide feedback for the program. | 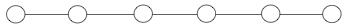 | | | | | |
| There should be more opportunities to receive feedback on individual performance. | 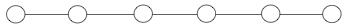 | | | | | |
| There should be more opportunities to teach. | 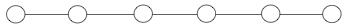 | | | | | |
| There should be more contact with patients. | 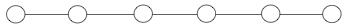 | | | | | |
| There should be more contact with students. | 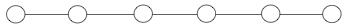 | | | | | |

17. Have you tried to make any changes to your workplace upon your return to SickKids from an international experience?

- Yes (go to question 17.1, 17.2)
- Yes, but I was unsuccessful (go to question 17.2)
- No

17.1 Please describe any changes you have made to your workplace upon your return to SickKids.

17.2 How has your international experience contributed to your desire to change your workplace at SickKids? *(Select the category that best reflects your experience)*

|  | Strongly Disagree (1) | Disagree  (2) | Neutral  (3) | Agree  (4) | Strongly Agree  (5) | N/A |
| --- | --- | --- | --- | --- | --- | --- |
| It has introduced me to new ideas or information. | 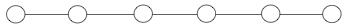 | | | | | |
| It has presented me with new technologies. | 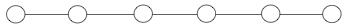 | | | | | |
| It has inspired a personal attitude change within myself. | 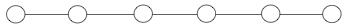 | | | | | |
| It has provided me with a new perspective to approach health care. | 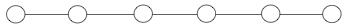 | | | | | |
| It has encouraged my existing beliefs about health care. | 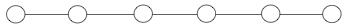 | | | | | |

18. Were you exposed to any innovative ideas or technologies during your international experience(s)?

- Yes (go to question 18.1, 18.2)
- No

18.1 Please describe any innovative ideas or technologies you were exposed to during your international experience(s).

18.2 How have you, or how might you apply these innovations or technologies at SickKids?

19. Do you think that your international work is valued by your colleagues?

- Absolutely
- Mostly
- Neutral
- Somewhat
- Not at all

20. Do you think that your international work is valued by senior administrators of the hospital?

- Absolutely
- Mostly
- Neutral
- Somewhat
- Not at all

21. Do you think that your international work is valued by the hospital foundation?

- Absolutely
- Mostly
- Neutral
- Somewhat
- Not at all

22. Are you more likely to continue working at SickKids due to the availability of international experiences?

- Absolutely
- Mostly
- Neutral
- Somewhat
- Not at all
- Not sure

23. Do you think that your international experience has had a positive impact on the SickKids brand back in Canada?

- Absolutely
- Mostly
- Neutral
- Somewhat
- Not at all
- Not sure

24. Please provide any additional reflections on your international experience(s) with SickKids, or your experience taking this questionnaire.

**Thank you for taking the time to complete this survey.**
