# Supplementary material for: Perceived reciprocal value of health professionals’ participation in global child health-related work
Source: Global Health. 2017 May 22;13:27. doi: 10.1186/s12992-017-0250-8 (PMC5441071; doi:10.1186/s12992-017-0250-8)
Supplement: Supplementary file 2 — List of countries visited. This file contains a complete list of countries where respondents had previously worked, as well as the number of respondents who worked at each location. (DOCX 72 kb) [file 12992_2017_250_MOESM2_ESM.docx]

**Appendix B: List of countries visited**

| **Country** | **Number of Participants** |
| --- | --- |
| Qatar | 139 |
| Ethiopia | 10 |
| Ghana | 6 |
| China | 5 |
| Tanzania | 4 |
| Bangladesh | 3 |
| Jamaica | 3 |
| Bahamas | 3 |
| India | 3 |
| United Arab Emirates | 3 |
| Caribbean | 2 |
| South Africa | 2 |
| Tanzania | 2 |
| Trinidad & Tobago | 2 |
| Nepal | 2 |
| Brazil | 2 |
| Peru | 2 |
| St Lucia | 1 |
| Barbados | 1 |
| Oman | 1 |
| Ecuador | 1 |
| Vietnam | 1 |
| Malaysia | 1 |
| Cameroon | 1 |
| Pakistan | 1 |
| Kenya | 1 |
| Myanmar | 1 |
| Gambia | 1 |
| Cambodia | 1 |
| Jordan | 1 |

Number of participants is not reflective of total participant number, as many participants visited more than one country.
