# Supplementary material for: Perceived reciprocal value of health professionals’ participation in global child health-related work
Source: Global Health. 2017 May 22;13:27. doi: 10.1186/s12992-017-0250-8 (PMC5441071; doi:10.1186/s12992-017-0250-8)
Supplement: Supplementary file 3 — Summary of participant responses to open-ended survey questions. This file contains a summary of all participant responses to open-ended survey questions. (DOCX 128 kb) [file 12992_2017_250_MOESM3_ESM.docx]

**Appendix C. Summary of participant reponses to open-ended survey questions.**

| **Question** | **Participant Responses** |
| --- | --- |
| Please describe any innovative ideas or technologies you were exposed to during your international experience(s). (n=29) | simulation technologies |
|  | Latest bells and whistles for research but no idea how to use them |
|  | New teaching methods (i.e.energizers) |
|  | different ways of securing PIVs |
|  | innovative equipment |
|  | multidisciplinary team training |
|  | methods of adult education. How creative people can be with limited resources |
|  | Robotics in the Operating room |
|  | Do more with less, whenever visiting developing countries. On the other hand, in European countries or in the USA, it was helpful to compare how they operate in comparison to Canada. |
|  | actually they were "old".....with no electricity or internet on many days of teaching we reverted back to the older methods |
|  | newborn passport in Tanzania to ensure all receive access to essential care |
|  | Bedside human milk analyzers; databases for computation of nutrient intakes; new statistical approaches to analyze data |
|  | Communication apps |
|  | keeping one day free in OR for thorough cleaning of operating rooms |
|  | use of web based communication for medical advice |
|  | Video conferencing, technologies of communication |
|  | IV acetaminophen |
|  | not ideas or technologies, but overall ways of providing clinical care, and organization of the flow of a system |
|  | community based delivery of care |
|  | I became aware of new cancer related syndromes related to specific populations, we also decided to implement new techiniques in countries with limited resources and to develop guidelines for these countries |
|  | Low-resource setting obstetrics for emergency management such as B-lynch procedure |
|  | greater focus on self-managed care |
|  | use on non invasive ventilation |
|  | ooo |
|  | online and interactive teaching techniques |
|  | models of care were quickly evolving to meet needs. In Canada, this seems to take forever |
|  | Robotics |
|  | different medications, neonatal technologies, low-resource methods for management of patients, many novel approaches for teaching/collabortions, pedogogy differences for teachig |
|  | Different Health Information System |
| How have you, or how might you, apply these innovations or technologies at SickKids? (n=23) | our team has discussed how to utilize some of the high fidelity simulation technologies that we were exposed to during our int'l experience |
|  | Sure! |
|  | i have tried role modeling the different ways of taping a PIV but it has not been sustained |
|  | no, d/t cost |
|  | Ongoing development of learning modules and education materials |
|  | Require capital 1st |
|  | By incorporating the best of what is seen outside, and by avoidance of the worst of what is also seen outside. |
|  | can use similar tool in Canada to promote optimal utilization of health care services |
|  | Purchased equipment; build a database; incorporated new statistical approaches to research |
|  | integrating similar technology to better enhance communication among SickKids staff |
|  | Can have designated hours per service per month for this purpose |
|  | I have not |
|  | we need this |
|  | no |
|  | small grants, regular videoconferences, exchange of material |
|  | I wouldn't--though I'm better equipped to apply this knowledge in the upcoming study design |
|  | currently in use |
|  | ooo |
|  | used online and QR codes to help families to access information |
|  | Lessen beaurocracy and power dynamics (especially physicians) to promote more rapid improvement and change |
|  | Not in my power to implement such techology |
|  | Less reliance on technology, greater understanding of non 'western' modalities of healing, |
|  | Compare functionality and design withour HIS and furture plans for HIS |
| Please describe any changes you have made to your workplace upon your return to SickKids. (n=30) | to help others be more multiculturally sensitive and open |
|  | Introduced new technology and forum for idea sharing |
|  | Provision for more culturally sensitive care especially on patients coming from the region I was in. |
|  | process improvement |
|  | Better planning for research course teachers |
|  | Encouraged colleagues to identify opportunities for mutually beneficial international partnerships |
|  | Increased predeparture training & debriefing for staff. |
|  | more aware of cultural diversities |
|  | Teaching methods and variations, recycling materials |
|  | formalizing a LGBTQ diversity committee |
|  | Apply same needs assessment tools to SickKids' programs |
|  | new opportunities for ongoing international teaching/mentorship |
|  | Ensuring curriculum includes cultural competency for all education programs |
|  | Collaboratively produced an improved Project Orientation |
|  | Acknowledging to superiors regarding inconsistent practices and communication among staffs (MD, RN, and allied health) within the same discipline (ie. Neonatal, surgery, ENT etc). |
|  | Resource education binder available to staff |
|  | motivated others to take part in international work at Sickkids |
|  | Improved focus on cultural competency |
|  | n/a |
|  | instigated cultural sensitivity in end-of-life care for our familie |
|  | Became resource person for patients coming from the countries/culture of my international work. |
|  | motivated me to seek teaching opportunities |
|  | increase recognition of value of bedside expertise |
|  | holistic and individual pt approach and family understanding |
|  | Grow a global health research program |
|  | Participation in videoconferences, developing collaborative research, stimulating participation in twinning activities (it does not always mean travelling!) |
|  | using different protocols for patient imaging |
|  | better, more practical teaching methods |
|  | translated more pamphlates and family resources |
|  | keep international partners in the communication loop more |
| Please provide any additional reflections on your international work experience(s) with SickKids, or your experience taking this questionnaire. (n=48) | A rewarding, worthwhile experience that helped me grow both personally and professionally. I am very grateful for the opportunity to have been involved in these projects. |
|  | I was honoured to have been chosen for this experience and valued my experiences while traveling with Sickkids. I think it definitely think it strengthened my skills and boosted my professional confidence. I look forward to future international experiences! |
|  | It was very frustrating to make recommendations that all agreed were needed but then minimal action on the part of the client |
|  | Would have been great to have some questions specifically directed at trainees, versus faculty (junior vs senior) |
|  | A significant challenge that has arisen with repetitive international assignments is garnering clearance from clinical coverage. My international work has been highly rewarding and is important work but I feel the weight of leaving my clinical area challenged to cover unit needs while I'm away on international work. |
|  | I have had 2 international experiences, one with Sick Kids and one outside of sick kids, they were dramatically different experiences, I think this survey should have been seperated into Sick Kids vs non-sick kids experiences because my responses would have been different for each |
|  | Thank you for this educational and amazing opportunity. I loved working with the Social Communication team in Qatar and seeing their determination and dedication was inspiring. |
|  | it was a cultural enlightment to be in Qatar...I enjoyed my stay |
|  | It's great to have the opportunity to serve and work internationally with SickKids. It benefits the organization and individual to provide more holistic and best practice care for patients and allows for feedback to further develop your professional skills |
|  | Good experience overall but difficult to keep up with workload at SickKids |
|  | Would love to continue to be given opportunities to go on short term trips related to my area of expertise. Unrelated, why was this survey not done using the sickkids survey tool or redcap? I thought the hospital policy was survey monkey was not allowed. |
|  | Lack of backfill in the my/colleagues role(s) whilst I was away caused stress to the members of the team who did not travel abroad. Workload for those who did not travel was heavier, at times "blamed" for shortage of staff whilst away. |
|  | I very much enjoyed the opportunity to share knowledge and worked with some wonderful, very motivated to learn health care providers during my placement in Qatar. However, the experience was marred by lack of communication and coordination resulting in a lack of clear objectives for the project I was assigned. There was little to no time provided to prepare content prior to arrival on the project. As a result I was required to prepare approximately 40 hours of lecture with 2 days prep time on site. End result, was there for 2 1/2 weeks and worked ~ 80 hours a week. No compensation in anyway for the additional work required to make the project successful. I would definitely be interested in doing international work again, however, not without better communication, coordination and clarification of objectives. |
|  | Perhaps I have a different perspective since 100% of my work is with international partners. I have little opportunity to apply what I learn while overseas to the hospital aside from the international partnerships I work on, although I do try to get involved in braoder hospital initiatives. |
|  | Questionnaire focused only on actual international travel and visits. The large portion of my contributions to international health are done while in Canada, through teleconferences, project planning, etc. I have been involved while in Canada with international initiatives in the Caribbean, UAE, Pakistan, Qatar, USA, etc. |
|  | Build capacity through mentoring and allow sufficient time to operationalize recommendations |
|  | This is a wonderful added oportunity for the right people to engage in. I always find I learn so much from the people I am in contact with in the countries I have worked with. I always maintain that I learn more than I teach. |
|  | Was a fantastic experience and feel fortunate to have been able to go over to Qatar and suppor the project. |
|  | My international work experience required a significant amount of unpaid and unrecognized work to develop almost a weeks worth of curriculum that I was sought out to provide. This unpaid work was completed both before and after I returned from my International experience on my own time. I was never able to ultimately institute the curriculum I created formally as the international team then regrouped and developed other priorities for teaching and education (addressing the 4 or 5 most significant pillars of need). This was immensely disappointing. Also, I believe the international team painted an overly positive and enthusiastic portrait of the seemingly supportive learning environment in Qatar. Many of the questions in this survey were somewhat difficulty to answer as the international experience I had did not significantly change or influence my practice. I did not have significant contact with those outside of the SickKids team while there. I found that educators and nursing staff from Qatar were hesitant to participate in curriculum development and the subsquent expected uptake of teaching the curriculum developed. I hope that this improved for other individuals who participated in this international experience later. I have learned to be more assertive and ask more questions and expect my time to be compensated prior to volunteering or accepting an international work experinece in the future. |
|  | Thank goodness for Skype; use of local phones while there or a long distance phone plan essential for safety |
|  | I feel that my particitaion was very minimal because I was with the wrong group of people during my visit to Qatar that did not appreciate my training, previous international experience and contributions |
|  | Extreme valuable experience to participate as a consultant with SickKids International |
|  | I think the biggest challenge (and also the biggest learning) related to the shifting terms of engagement between SickKids and HMC. Also, at times it felt that we were doing things just to tick off the box that it was done, and therefore not often able to modify a 'deliverable' in a way that might have actually made a more meaningful contribution |
|  | I am looking forward to other internation experiences when they become available in other countries. |
|  | Need to work harder to bridge expectations of various stakeholders. Disjointed vision from project managers, clinicians, funders, etc..Interventions delivered and evaluated in piecemeal fashion. Sustainability should be emphasized. |
|  | it is a wonderful opportunity for staff at Scikkids, a privledge.... |
|  | There was a lot of problems with Bloorview who were there in their capacity - this could have been much better coordinated which would have generated less of a confrontational atmosphere. |
|  | Look forward to SickKids expanding in their international work...have had many colleagues who are interested in this field |
|  | Having provided health care in First Nations communities for years I was struck by the unpreparedness and at times total insensitivity of well meaning Sick Kids staff. Eg. Disregarding a Qatari physician's focus on the child's well-being; being unfamiliar with how medical education is provided and inadvertently insulting a staff physician by ignoring his attempt to answer questions and focusing on the trainees as we do here, inadverntently including the Qatari staff on a Sick Kids email describing a particular unit's lack of medical understanding and ability, recommending that staff nurses "speak up" and advocate for their patients in an environment where women do not have a voice. |
|  | Loved all my international experiences! So rewarding to make such an impact. |
|  | Wasn't in a developing country, which would have had a bigger impact on me. I think SKI and Hamad have overestimated the value of the Qatar project, slhtough i do think that positive changes took place. One issue for me was no opportunity to actually get to know any Qatari's, this was really an ex-pat related experience. Brought a lot of money into the organization, i gather, which is good... |
|  | more personal compensation would be nice, for example, days back for travel time or payment for extra work when doing it on your own time to prep |
|  | I thoroughly enjoyed the experience - would go again if given the opportunity - I think there is great value to both SickKids and the outreach community in providing this type of opportunity to those communities - sharing of expertise is essential in promotion of global child health - I think there should be more fund raising targeting this type of sharing of expertise to the third world - many of these personal skills I had full confidence in prior to the international experience |
|  | What is Sickkids "brand" |
|  | As many of my colleagues, I feel that the international work (I mean humanitarian) is essentially a personal commitment, not a Sickkids way of thinking. However, Sickkids offers unique tools (videoconference, protected time) that allow us to commit to these important activities |
|  | Positve overall. More support prior to going would have been helpful and after coming home there was no follow up from SKI and I had feedback but didn't know who I should have spoken to about it. Better communication from my manager to my team that I was going would have perhaps made the experience more positve. There was no encouragememt from my manager prior to going and my team mates seemed not to care. More opportunity for staff who have worked at SK a long time to paritciate in these trips across all diciplines not just medical staff would be wonderful. Some transparency about how staff are chosen and why they return mulitple times would also have been helpful to the team members left behind. |
|  | I'm a practice midwife in the community in Ontario and a post-doc in epidemiology. I have other international experience before coming to HSCtherefore my recent short stint gave me some new experiences but I was well-aware of existing resources and limitations in maternity care, teaching, etc. that's why I responded neutral to a lot of questions. As well, I don't provide clinical care at HSCso many questions regarding patients don't apply. |
|  | Opportunity to participate in the project for a longer period would have made my contribution more impactful. Alternatively, more opportunity to receive a formal handover from the provider there before me regarding the short term goals of the program would have been beneficial. Eliciting more buy-in from frontline stakeholders at the health care facility would have been beneficial. |
|  | I would love to work for SKI. |
|  | Great opportunity to meet and work with SickKids colleagues that I don't routinely work with day to day. I learned so much from and about them. I valued the opportunity to teach or to do assessment there and never took for granted the opportunity. I developed lasting friendships with part of the Qatar medical team and continue to correspond with them. I felt very proud of the accomplishments and would welcome the opportunity to see whether the competencies are sustained over time. |
|  | It gave me the opportunity to meet, work with and learn from other SickKids staff who I would otherwise never have met. The interprofessional collaboration initiatives we started in Qatar were a wonderful experience for us as SIckKids staff and for the clinicians we were working with at HMC. I decided to go back to school - Masters in Health Management - due to my experience with SKI. |
|  | Felt the experience was valued and well supported by SKI, and formally supported by my department however not informally supported through verbal support or action by my department. |
|  | would like to be part of the International Prgram again, truly enjoyed the experience |
|  | Good luck with your survey. |
|  | I have had international experience for over a decade, so honestly I didn't add a lot to my own development. I do like teaching and providing my experience to people abroad. That part I enjoy the most. Whenever SKI will need me, I will try to make this work. |
|  | i have done far more international work outside of sickkids on behalf of other organizations such as the RCPSC |
|  | an amazing life experience |
|  | Thankful that I was chosen and had the opportunity to participate in this project |
